# Oral delivery of double-stranded RNAs induces mortality in nymphs and adults of the Asian citrus psyllid, *Diaphorina citri*

**DOI:** 10.1371/journal.pone.0171847

**Published:** 2017-03-10

**Authors:** Diogo Manzano Galdeano, Michèle Claire Breton, João Roberto Spotti Lopes, Bryce W. Falk, Marcos Antonio Machado

**Affiliations:** 1 Laboratório de Biotecnologia, Centro de Citricultura Sylvio Moreira, Instituto Agronômico de Campinas, Cordeirópolis, São Paulo, Brazil; 2 Instituto de Biologia, Universidade Estadual de Campinas, Campinas, São Paulo, Brazil; 3 Escola Superior de Agricultura Luiz de Queiróz, Universidade de São Paulo, Piracicaba, São Paulo, Brazil; 4 Plant Pathology Department, University of California Davis, Davis, California, United States of America; United States Department of Agriculture, UNITED STATES

## Abstract

The Asian citrus psyllid (ACP), *Diaphorina citri* Kuwayama, is one of the most important citrus pests. ACP is the vector of the phloem-limited bacteria *Candidatus* Liberibacter americanus and *Candidatus* Liberibacter asiaticus, the causal agents of the devastating citrus disease huanglongbing (HLB). The management of HLB is based on the use of healthy young plants, eradication of infected plants and chemical control of the vector. RNA interference (RNAi) has proven to be a promising tool to control pests and explore gene functions. Recently, studies have reported that target mRNA knockdown in many insects can be induced through feeding with double-stranded RNA (dsRNA). In the current study, we targeted the *cathepsin D*, *chitin synthase* and *inhibitor of apoptosis* genes of adult and nymph ACP by feeding artificial diets mixed with dsRNAs and *Murraya paniculata* leaves placed in dsRNAs solutions, respectively. Adult ACP mortality was positively correlated with the amount of dsRNA used. Both nymphs and adult ACP fed dsRNAs exhibited significantly increased mortality over time compared with that of the controls. Moreover, qRT-PCR analysis confirmed the dsRNA-mediated RNAi effects on target mRNAs. These results showed that RNAi can be a powerful tool for gene function studies in ACP and perhaps for HLB control.

## Introduction

The Asian citrus psyllid (ACP), *Diaphorina citri* Kuwayama (Hemiptera: Liviidae), is a phloem-feeding insect and the most economically important pest of citrus, mainly because it is the vector for the causal agents of huanglongbing (HLB), *Candidatus* Liberibacter asiaticus and *Candidatus* Liberibacter americanus [1,2]. HLB occurs throughout the citrus-growing regions of Asia, Africa and the Americas and is the most important disease affecting citrus worldwide, resulting in tree decline, reduced fruit quality and tree death [3–5]. Both ACP adults and nymphs can transmit the bacteria, and when it is acquired by nymphs, the bacteria can be retained and transmitted after the emergence of adults, suggesting that the pathogen multiplies and circulates in the ACP [6]. Moreover, the bacteria may be transmitted at low rates from infected females transovarially to their progeny [7] and from infected males to uninfected females during mating [8].

To control *D*. *citri* populations, citrus growers use several different insecticides [9]. However, the indiscriminate and continuous use of these insecticides can lead to pest resistance, which invariably leads to increased costs in production [10]. A main challenge for researchers has been to develop efficient strategies for *D*. *citri* control with low environmental impact, such as the use the ectoparasitoid *Tamarixia radiata* (Hymenoptera: Eulophidae) [11] as a natural predator [12], entomopathogenic fungi [13] and recently RNA interference (RNAi) [14–16].

RNAi is a powerful tool for studying functional genomics in eukaryotes, including insects [17]. The discovery of double-stranded RNA (dsRNA)-mediated gene-specific silencing in the nematode *Caenorhabditis elegans* [18] enabled dsRNA-mediated RNAi to be employed towards various crop insects to silence specific genes [19].

The first step for successful RNAi in insects is to identify a convenient and reliable method for the delivery of dsRNA to the target gene. Microinjection, soaking and feeding are typically adopted to deliver dsRNA into insects [20]. Successful target mRNA knockdown effects via artificial feeding of dsRNAs have been reported in crop pest insects, including the potato/tomato psyllid (*Bactericera cockerelli*), whiteflies (*Bemisia tabaci*), western corn rootworm (*Diabrotica virgifera virgifera*), pea aphid (*Acyrthosiphon pisum*), cotton bollworm (*Helicoverpa armigera*), oriental fruit fly (*Bactrocera dorsalis*), beet armyworm (*Spodoptera exigua*), and the brown planthopper (*Nilaparvata lugens*) [21–29]. However, it is difficult use artificial feeding of dsRNAs during the juvenile stages of some insects; thus, an efficient system was developed to silence genes of whitefly nymphs through leaf-mediated dsRNA feeding [30]. Moreover, some studies have demonstrate that the ineffectiveness of oral delivery of RNAi among insect species is attributable to the rapid degradation of dsRNA, which is probably induced by nuclease enzymes in the gut lumen [31,32] or in the saliva [33], thus for these insect species, the better delivery method of dsRNA is injection of naked dsRNA into the body cavity [34].

The next step for successful to RNAi-based insect control is identify potential targets genes involved in essential biological processes [35]. The enzyme cathepsin D is a lysosomal aspartic proteinase located in the posterior midgut of hemipterans that is responsible for intracellular and extracellular protein digestion and is involved in metamorphic events in insects [36]. Injection of *cathepsin D* dsRNA into *Bombyx mori* larvae caused metamorphic defects, such as larval-pupal transformation arrest and programmed cell death inhibition [37]. Insect chitin synthases are the main enzymes for the development of the trachea, cuticle and midgut [38]. Chitin synthases are encoded by two genes, *chitin synthase A* and *B*. *Chitin synthase A* genes are expressed in tracheal, epidermal and ectodermal cells, while *chitin synthase B* genes are expressed in gut epithelial cells involved in the production of the peritrophic matrix of the insect midgut [39]. In a demonstration of the effects of RNAi in *S*. *exigua*, larvae fed *Escherichia coli* expressing *chitin synthase A* dsRNA exhibited lethal alterations in growth and development [27]. Inhibitor of apoptosis proteins were first identified for their role in the regulation of apoptotic machinery. *Inhibitor of apoptosis* genes have also been identified in insects, yeast and mammals, and these genes have been observed to play a role in various processes, such as intracellular signalling, ubiquitination, homeostasis, cellular morphogenesis and cellular division [40]. dsRNA topically applied targeting an *inhibitor of apoptosis* gene in *Aedes aegypti* was able to kill these mosquitoes [41].

In the present study, we explored the effects of RNAi in adult and nymph *D*. *citri* which were fed gene-specific dsRNAs targeting *cathepsin D*, *chitin synthase* and *inhibitor of apoptosis* via an artificial diet and through plant leaves. We showed that both dsRNA delivery methods are efficient in silencing genes of ACP, increasing the mortality rate over time and down-regulating target genes.

## Materials and methods

### Insects and plants

ACPs were reared in *Citrus macrophylla* in mesh cages maintained at 25 ± 2°C under a 14:10 h (light:dark) photoperiod and 60 to 70% relative humidity (RH) at the Contained Research Facility (CRF) of the University of California, Davis (CA, USA; http://crf.ucdavis.edu/). Teneral adult ACPs and mated females were used for the feeding assays on artificial diet and in *Murraya paniculata* (L) Jack (Rutaceae) leaflets, respectively.

### RNA isolation and cDNA synthesis

Total RNAs were isolated from a pool of ten live ACPs using ZR Tissue and Insect Microprep^™^ (Zymo Research, Irvine, CA, USA, catalogue no. D6015), and the genomic DNA was removed from the samples with a DNAse I Set (Zymo Research, Irvine, CA, USA, catalogue no. E1010) according to the manufacturer’s protocol. The concentration and purity were determined using a NanoDrop ND 8000 spectrophotometer (NanoDrop Technologies, Wilmington, DE, USA). cDNA synthesis was performed using iScript^™^ Reverse Transcription Supermix (Bio-Rad^®^, Hercules, CA, USA, catalogue no. 170–8841) according the manufacturer’s protocol.

### Identification, primer design and amplification of target genes

To identify the *cathepsin D*, *chitin synthase* and *inhibitor of apoptosis* genes of ACP, annotation of the *D*. *citri* transcriptome sequences was performed by screening the data bank Psyllid.org (http://psyllid.org/download) based on amino acid sequences of insects deposited in the NCBI (*Acyrthosiphon pisum*, *Aedes aegypti*, *Anopheles gambiae*, *Apis florea*, *Apis mellifera*, *Bombus impatiens*, *Bombus terrestris*, *Drosophila melanogaster*, *Drosophila pseudoobscura*) using BlastX. Primers and probes were designed using the PrimerBlast tool (http://www.ncbi.nlm.nih.gov/tools/primer-blast/) (Table 1). The PCR product corresponding to GFP sequence was amplified from plasmid pJL24. PCRs were performed using standard procedures (Sambrook, 2012) with GoTaq^®^ DNA Polymerase (Promega Corporation, Madison, WI). The fragments were analysed on 1% agarose gels containing 1% SYBR^®^ Safe DNA (Invitrogen^®^). The amplicon sequences were confirmed by sequencing.

**Table 1:** List of primers and probes used in this study.

Primer name	Forward (5’-3’)	Reverse (5’-3’)	Probe (5’-3’)
**PCR**			
Cathepsin D	AAACAGACCTGGGAAACGCT	GCATCTTGTCCAAGTTGTCGC	
Chitin Synthase	AACTTTGGTCGAGACAAGCAG	TCCCAATAACCGCAGGAC	
Inhibitor of Apoptosis	TTTCGGTATCCTCGCAGATG	AGCTCTGCATGGTGTTTGATG	
GFP	TACGGCGTGCAGTGCTTCA	CGTCCTCGATGTTGTGGCG	
**qRT-PCR**			
Cathepsin D	GCTAATGGACCTGCCAAAGT	TTTGTTGCAGTGAGGGTGAAG	TCGACGCTAGTCATCAGAGCTTCCA
Chitin Synthase	TCAGCATGGCGGGTTAAG	CTCCGCGGAATGACATGAATA	TGCCTTCGTGTCATTCCTCAAGATCC
Inhibitor of Apoptosis	GGCTGAACTGTCTCCATTCTAT	GAACTCAGGTTCTGTGTCTTCT	TGCTTGTTTACCTATCTGCCCACTCC
Actin	TGACATCAAGGAGAAGCTGTG	GTCGGGAAGTTCGTAGGATTT	TCGCCCTGGACTTTGAACAGGAAA

### Double-stranded RNA preparation

The dsRNAs were synthesized *in vitro* with the MEGAscript RNAi kit (Ambion, catalogue no. AM1626) using target genes and GFP PCR products as templates. The T7 sequence (5’ TAATACGACTCACTATAGGGAGA 3’) was placed in front of both the forward and reverse primers which were used for PCR amplification of the template for dsRNA synthesis. PCR of *cathepsin D*, *chitin synthase*, *inhibitor of apoptosis* and GFP coding regions was performed using GoTaq^®^ Colorless Flexi Buffer 5X (Promega), 25 mM MgCl_2_, 10 mM dNTPs, 10 μM forward primer plus T7 promoter, 10 μM reverse primer plus T7 promoter, 5 U/μL GoTaq^®^ DNA Polymerase and 1 μL cDNA. PCR products were purified according to the QIAquick PCR Purification Kit (Qiagen, CA, USA).

*In vitro* transcription was performed using purified DNA (1 μg), 10X reaction buffer, ribonucleotide (ATP, GTP, CTP, UTP) and enzyme T7. The reaction was incubated at 37°C overnight. The template DNA was removed using a TURBO DNA-free kit (Ambion, TX, USA). The dsRNAs were precipitated by adding 30 μL lithium chloride precipitation solution (7.5 M lithium chloride, 50 mM EDTA) and 30 μL nuclease-free water. The samples were incubated for 1 h at -20°C and centrifuged at 4°C for 15 min at 16,000 g. The supernatant was removed, and the pellets were washed once with 1 mL 70% ethanol. The ethanol was removed, and the dsRNA was eluted in 20 μL nuclease-free water. The dsRNA quality was monitored using agarose gel electrophoresis, and the concentration was determined using NanoDrop.

### Bioassays for feeding-based RNAi studies using artificial diets

Teneral adult ACPs were allowed to feed on an artificial diet solution consisting of 15% (w:v) sucrose and food dyes (0.1% green and 0.4% yellow) (McCORMICK & CO). An aliquot of the artificial diet (100 μL) was pipetted onto the parafilm and another layer stretched on top of the clear plastic vial (25 mm X 45 mm) to form a sachet and to ensure the liquid diet was distributed evenly. Feeding assays were performed at room temperature in a 14:10 h (light:dark) photoperiod and 60 to 70% RH, and the plastic vials were placed 1.20 m from the light source. For the mortality evaluations, *cathepsin D*, *chitin synthase* and *inhibitor of apoptosis* dsRNA concentrations (200, 500, 1000 ng.μL^-1^) were standardized and diluted in the artificial diet and used for feeding assays. Each replicate consisted of 30 individuals in 3 plastic vials (10 individuals in each vial), and three replicates were analysed. GFP dsRNAs at the same concentrations in feeding solutions of 15% sucrose were used as controls for each experiment. After the teneral adult ACPs were fed for 120 h, total RNAs were isolated from a pool of three adult ACPs and used for cDNA synthesis as described above.

To test the stability of dsRNAs in the artificial diet solution, 50 μL of each solution was collected after the 5^th^ day of feeding. The artificial diet solutions were diluted in distilled water (1/10), and the dsRNAs were observed in 1% agarose gels.

### Bioassays for feeding-based RNAi studies using *Murraya paniculata* leaflets

A method previously described to induce RNAi effects in whiteflies [30] was used to evaluate the RNAi effects on *D*. *citri* nymph survival with modifications. Briefly, one leaflet was cut from an *M*. *paniculata* plant and placed in a 1.5 mL microtube containing distilled water for one day to recover. The microtube with the leaflet was transferred to a Falcon flask (50 mL) and covered with a piece of anti-aphid mesh tightly held with a rubber band. One mated female adult *D*. *citri* was released onto the leaf through a hole in the middle of the Falcon flask wall, allowed to oviposit for 24 h and then removed. The leaflet was then transferred to a 1.5 mL microtube containing 0.5 mL of each dsRNA (*cathepsin D*, *chitin synthase*, *inhibitor of apoptosis* and GFP) at the concentration 500 ng.μL^-1^ or distilled water. The microtube with the leaflet was returned to the Falcon flask (50 mL) and covered as described above. The assays were performed at room temperature in a 14:10 h (light:dark) photoperiod and 60 to 70% RH, and five replicates were used for each treatment. The solution in the microtube was replenished every four days, and the percentage of nymph survival was counted on the 5^th^, 7^th^, 9^th^ and 11^th^ days. After 11 days, total RNAs were isolated from a pool of three 3^rd^ instar nymphs and used for cDNA synthesis as described above.

The stability of dsRNAs in the tubes and leaves was assessed on the last day of the assay. dsRNAs from the tubes were collected on the 11^th^ day of the feeding assay described above. To detect the dsRNAs in leaves, a piece of leaf (0.05 g) was collected and immediately frozen in liquid nitrogen. Total RNA was isolated from leaf tissues. Primers for dsRNA synthesis along with the RNA samples were heated in boiled water for 5 min and quenched on ice. Then, the RNA was reverse transcribed and cDNA was used for RT-PCR [30]. Both the dsRNA solution from the tubes and PCR products of dsRNAs from plant leaf tissues were analysed by electrophoresis in 1% agarose gels to examine the size and integrity of dsRNAs.

### Quantitative Reverse-Transcription Polymerase Chain Reaction (qRT-PCR)

The effect of dsRNAs on target mRNA levels was quantified by qRT-PCR using iTAQ^™^ Universal Probes Supermix (Bio-Rad^®^, catalogue no. 172–5131). To avoid RT-PCR artefacts resulting from the input dsRNAs, primers and probes for qRT-PCR were designed to detect target mRNAs by amplifying sequences located outside of the input dsRNA sequences (Table 1). The primer design was performed using *RealTime qPCR Assay* (https://www.idtdna.com/primerquest/Home/Index). The *D*. *citri* actin gene was used as an internal control. The stability of *D*. *citri actin* gene from feeding assays in leaflet plant and artificial diet analyzed in GeNorm showed that the M value data from *actin* were 0.05 and 0.07, respectively, showing that this gene was stable in the experimental conditions analyzed indicating be useful as an internal control for normalization (S1 Fig). The reaction mixture contained 10 μL iTAQ Universal Probes Supermix (2X), 0.18 μL forward and reverse primers (5 μM), 0.25 μL fluorogenic probe of mRNA targets (1 μM), 0.25 μL fluorogenic probe of *actin* mRNA (1 μM), and 6 μL cDNA and nuclease-free water in a final reaction volume of 20 μL. The standard amplification curves of *actin* and target mRNA were generated by diluting the amplified fragments as templates to verify the amplification efficiency of each primer set under the same amplification conditions. The amplification reactions were performed in the CFX96 Touch^™^ Real-Time PCR Detection System (Bio-Rad^®^), and the reaction conditions were 3 min at 95°C followed by 40 cycles of 95°C for 10 s and 59°C for 30 s for the annealing of primers and probes. GFP dsRNA was used as a control treatment.

Relative gene expression was evaluated using the 2^-ΔΔCT^ method. The average value and standard error value (SE value) of the 2^-ΔΔCT^ value in treatment and control groups were calculated separately, and the statistical analyses were performed to compare these two groups. Reactions were set-up in 96-well Microseal PCR plates (Bio-Rad^®^, Hercules, CA, USA) in triplicate.

### Statistical analysis

Experimental data from dsRNA feeding assays and target mRNA knockdown expression were evaluated using one-way ANOVA with Tukey’s Test.

## Results

### ACPs mortality induced by dsRNAs on artificial diet

**Fig 1:**
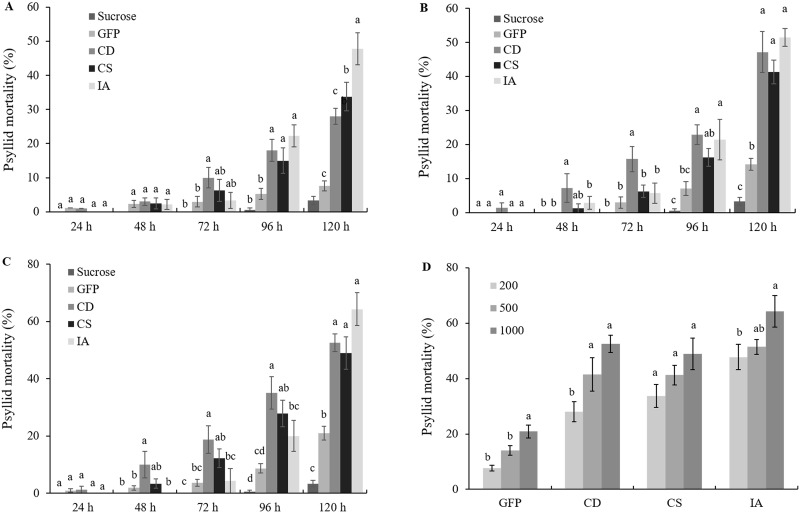
Mortality rate of psyllids fed artificial diets. Mortality rates by feeding 15% sucrose artificial diets containing dsRNAs of GFP, *cathepsin D*, *chitin synthase* and *inhibitor of apoptosis* at (A) 200 ng.μL^-1^, (B) 500 ng.μL^-1^ and (C) 1000 ng.μL^-1^ over time. Different letters indicate statistically significant differences in psyllid mortality rates between different treatments at the same time points (P<0.05). (D) Psyllid mortality induced by increasing concentrations 200, 500 and 1000 ng.μL^-1^ of *cathepsin D*, *chitin synthase* and *inhibitor of apoptosis* and GFP dsRNAs after 120 h of the feeding assay. Statistical differences are also shown by different letters between different concentrations for the same dsRNA at the same time points CD: *cathepsin D* dsRNA; CS: *chitin synthase* dsRNA; IA: *inhibitor of apoptosis* dsRNA.

To determine the optimal concentration of dsRNA for ACP mortality, a series of dsRNA concentrations (200, 500 and 1000 ng.μL^-1^) were tested for RNAi by feeding on artificial diet for five days. On the fifth day, ACP mortality was less than 5% when they were fed only with 15% sucrose, demonstrating the efficacy of the feeding system. The three target dsRNAs evaluated here exhibited significantly increased mortality compared with that observed for the GFP dsRNA control for all concentrations tested. Moreover, the ACP mortality increased over time, reaching maximum values at 120 h post-treatment (Fig 1A–1D). The artificial diet containing the *inhibitor of apoptosis* dsRNA at 200 ng.μL^-1^ resulted in 22% ACP mortality after 96 h and increased to 48% after 120 h. These percentages were significantly increased compared with those observed for the GFP dsRNA control and for other dsRNAs. The concentration of 200 ng.μL^-1^ of *cathepsin D* dsRNA caused 10, 18 and 28% mortality after three, four and five days, respectively. Moreover, the mortality resulting from *chitin synthase* dsRNA was significantly increased compared with that of GFP dsRNA after 96 and 120 h (15 and 34%, respectively) (Fig 1A). At 120 h post-treatment, *inhibitor of apoptosis*, *cathepsin D* and *chitin synthase* dsRNA at 500 ng.μL^-1^ resulted in 51, 47 and 41% ACP mortality, respectively (Fig 1B), and 1000 ng.μL^-1^ dsRNA produced similar results (64, 52 and 49% mortality, respectively) (Fig 1C). These results demonstrated that *inhibitor of apoptosis* dsRNAs induced the most potent ACP lethality among the three candidate dsRNAs examined in this study. Concentrations of the GFP dsRNA control (500 and 1000 ng.μL^-1^) caused significantly increased mortality compared with the 15% sucrose control after 120 h (Fig 1B and 1C). It is possible that high concentrations of dsRNAs are able to initiate off-target or RNAi pathway saturation effects deleterious to ACPs [42–44].

The mortality of *D*. *citri* exhibited a positive correlation with the concentrations of target genes and GFP dsRNA treatments (Fig 1D). All of the test and GFP dsRNAs caused higher mortality at 1000 ng.μL^-1^ compared with lesser concentrations for each dsRNA tested.

After five days, the dsRNAs were detected in 15% sucrose artificial diets, demonstrating the stability these molecules and indicating that feeding dsRNAs via artificial diets is a good approach to selecting RNAi targets for *D*. *citri* (S2 Fig).

### Nymph mortality rates induced by dsRNA uptake through *M*. *paniculata* leaves

**Fig 2:**
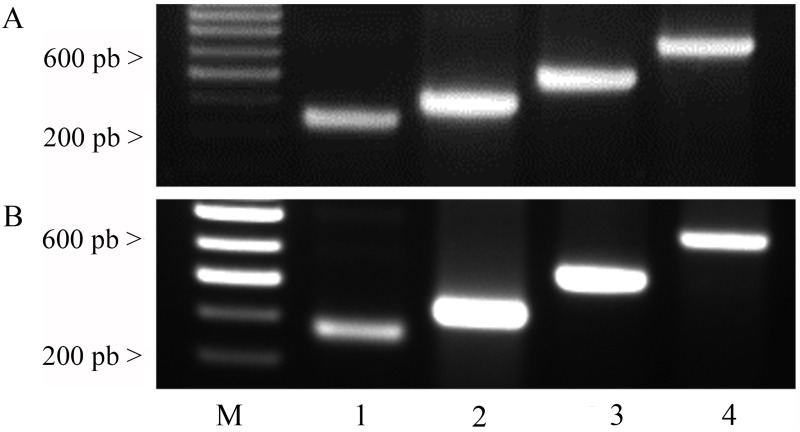
dsRNA stability in solutions and in plant leaves. Stability of *cathepsin D*, *chitin synthase*, *inhibitor of apoptosis* and GFP dsRNAs from solutions in which *M*. *paniculata* leaflets were soaked (A) and in leaves (B) on the 11^th^ day of the feeding assay were analysed by agarose gel electrophoresis. CD: *cathepsin D* dsRNA; CS: *chitin synthase* dsRNA; IA: *inhibitor of apoptosis* dsRNA.

A method was developed to silence ACP genes by dsRNA feeding through plant leaflets. The uptake of the dsRNA by the *M*. *paniculata* leaflet and its stability off in both the microtubes and the plant tissues were assessed on the last day of the feeding assay. The results showed that all dsRNAs, *cathepsin D*, *chitin synthase* and *inhibitor of apoptosis*, and GFP were stable in the tubes (Fig 2A) and in the leaves during the 11-day experiment (Fig 2B). Hence, this approach of delivering dsRNAs via cut leaves was subsequently used to target mRNA knockdown to silence genes in *D*. *citri*.

**Fig 3:**
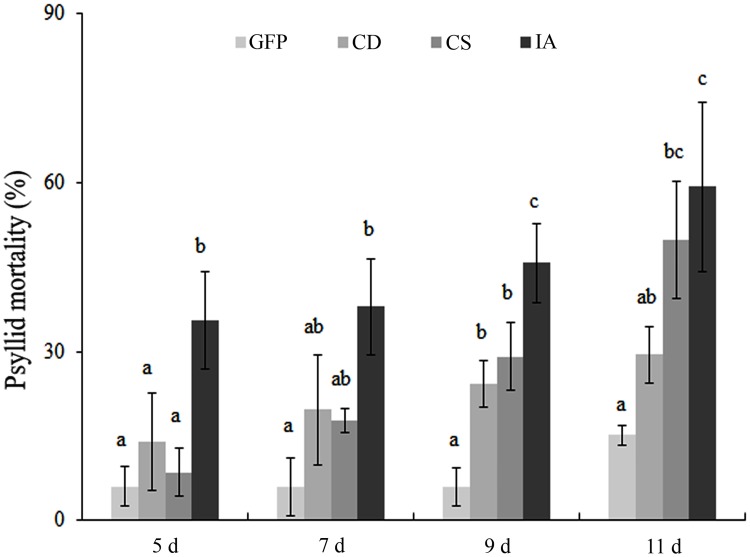
Mortality rate of *D*. *citri* nymphs fed dsRNAs through *M*. *paniculata* leaves. Survival rate of *D*. *citri* nymphs by the feeding of dsRNAs of GFP, *cathepsin D*, *chitin synthase* and *inhibitor of apoptosis* through *M*. *paniculata* leaves over time. Different letters indicate statistically significant differences in psyllid mortality rates between different treatments at the same time points (P<0.05). CD: *cathepsin D* dsRNA; CS: *chitin synthase* dsRNA; IA: *inhibitor of apoptosis* dsRNA.

The effects of *cathepsin D*, *chitin synthase* and *inhibitor of apoptosis* mRNA knockdown on ACP nymph survival were appraised by inserting detached *D*. *citri* egg-infested leaflets in a solution of dsRNA directed against each of three targets and the GFP control. *M*. *paniculata* leaflets containing *D*. *citri* eggs were placed in water, dsRNA solutions of each target and GFP at 500 ng.μL^-1^. Assessments of the nymph survival rate were performed at the beginning of the 5^th^ day because all viable eggs were already hatched. Thus, the evaluations were performed on the 5^th^, 7^th^, 9^th^ and 11^th^ days after placing the leaflets in the dsRNA solutions. After 11 days, the nymph mortality in leaflets in the water treatment was approximately 28% (data not shown), demonstrating the efficacy of the feeding system. Thus, the experimental averages were corrected by division of the nymph survival observed for each dsRNA treatment by nymph survival obtained in the water control. *Cathepsin D* dsRNA increased the nymph mortality from 14 to 29% at 5 and 11 days, and a difference was observed only on the 7^th^ day compared with the GFP dsRNA (Fig 3). Furthermore, *chitin synthase* dsRNA demonstrated significant increase of 21 and 50% in the nymph survival at 7^th^ and 11^th^ days, respectively. Interestingly, nymphs rapidly died when fed leaflets in *inhibitor of apoptosis* dsRNA solution on the 5^th^ day, exhibiting 36% survival that was increased to 60% after 11 days. These results demonstrate that *inhibitor of apoptosis* dsRNA induces the most potent lethality in nymphs fed RNAi among the three candidate genes examined in this system.

### Confirmation of target mRNA knockdown by dsRNA from artificial diet and *M*. *paniculata* leaves

**Fig 4:**
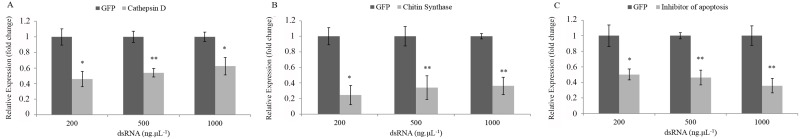
Knockdown of endogenous psyllid mRNAs by dsRNA feeding on artificial diets after 120 h. The dsRNAs were added to the artificial diets at concentrations of 200, 500 and 1000 ng.μL^-1^, and total RNAs were isolated from live psyllids after 120 h of feeding. GFP dsRNA-fed *D*. *citri* served as controls for each experiment. (A) Accumulation of *cathepsin D* mRNAs in whole psyllids fed *cathepsin D* dsRNAs at 200, 500 and 1000 ng.μL^-1^. (B) Accumulation of *chitin synthase* mRNAs in whole psyllids fed *chitin synthase* dsRNAs at 200, 500 and 1000 ng.μL^-1^. (A) Accumulation of *inhibitor of apoptosis* mRNAs in whole psyllids fed *inhibitor of apoptosis* dsRNAs at 200, 500 and 1000 ng.μL^-1^. Significant differences in relative expression were evaluated between test dsRNAs and GFP dsRNA at the same concentrations. A single asterisk indicates P<0.05, and double asterisks indicate P<0.01.

To determine whether the dsRNAs acquired orally from artificial diets and through *M*. *paniculata* leaves induced target mRNA knockdown, quantitative real-time PCR was used to assess target mRNA levels in ACP. Teneral adults' ACPs were collected 120 h after they were fed artificial diets at 200, 500 and 1000 ng.μL^-1^ dsRNAs, and total RNAs were extracted from live ACPs. After exposure to *cathepsin D* dsRNA for five days, the relative expression level of the target gene in *D*. *citri* was reduced to 46, 52 and 64% on 200, 500 and 1000 ng.μL^-1^ dsRNAs, respectively (Fig 4A). When ACPs were continuously fed with *chitin synthase* dsRNAs at 200, 500 and 1000 ng.μL^-1^, mRNA reductions of 76, 66 and 48% were noted five days post-feeding, respectively (Fig 4B). Moreover, the mRNA levels of *inhibitor of apoptosis* of *D*. *citri* were reduced to 50, 46 and 36% on 200, 500 and 1000 ng.μL^-1^ dsRNAs, respectively (Fig 4C).

**Fig 5:**
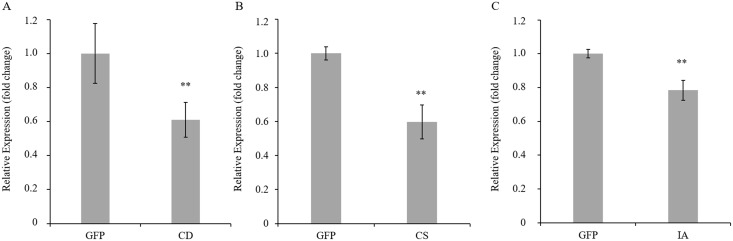
Knockdown of endogenous psyllid mRNAs by dsRNA fed *M*. *paniculata* leaves after 11 days. *M*. *paniculata* leaflets were soaked in dsRNA solutions at a concentration of 500 ng.μL^-1^, and total RNAs were isolated from live third-instar nymphs after 11 days of feeding assays. GFP dsRNA-fed *D*. *citri* served as controls for each experiment. Expression levels of *cathepsin D* (A), *chitin synthase* (B) and *inhibitor of apoptosis* (C). Significant differences in relative expression were evaluated between test dsRNAs and GFP dsRNA at the same concentrations. Double asterisks indicate P<0.01. CD: *cathepsin D*; CS: *chitin synthase*; IA: *inhibitor of apoptosis*.

ACP third-instar nymphs were collected 11 days after feeding dsRNAs of each gene through *M*. *paniculata* leaves, and total RNAs were extracted from live nymphs. After nymphs were fed for 11 days on dsRNAs via leaflets, *cathepsin D*, *chitin synthase* and *inhibitor of apoptosis* expression levels were reduced to 61, 59 and 76%, respectively, and all genes were decreased significantly compared with those in the control (Fig 5A–5C). These results demonstrate that feeding dsRNA via artificial diets and *M*. *paniculata* leaves caused the target mRNA knockdown of *D*. *citri*.

## Discussion

Sequence-specific suppression of gene expression by RNAi offers great opportunities for insect science, particularly for gene function analysis, pest management and the reduction of diseases caused by pathogens. A suitable delivery system for dsRNAs can be a major limitation of RNAi studies for insects [20,34,45–48]. For sap-sucking insects, such as sharpshooters, aphids, whiteflies and psyllids, dsRNA delivery methods have been demonstrated [29,42,49,50]. In the present study, we demonstrated the induction of specific RNAi effects in ACP by oral acquisition of dsRNA from artificial diets and *M*. *paniculata* leaves.

The artificial diet containing 15% sucrose and food colouring used here was developed for screening dsRNAs for targeting the potato/tomato psyllid *B*. *cockerelli* [42], and we adapted this method to evaluate the RNAi effects in *D*. *citri*. This system was used to evaluate three RNAi targets via dsRNA delivery by feeding, and we demonstrated its efficacy because the mortality of psyllids in the sucrose control treatment was less 5% even after 120 h of feeding. Moreover, dsRNAs were stable in the artificial diet solution on the last day of the assay, indicating that this approach can be useful for screening RNAi inducers against *D*. *citri*. Similar results were observed on artificial diets containing *V-ATPase* dsRNAs for RNAi in *B*. *dorsalis* [26], *N*. *lugens* [51] and *B*. *cockerelli* [42] at 6, 24 and 96 h post-feeding, respectively. However, dsRNAs present in artificial diets can be degraded by nucleases in the salivary secretions of some insects. For example, aphids feeding on dsRNA of GFP for 84 h resulted in dsRNA degradation, indicating that salivary secretions from the aphids while feeding caused the degradation of dsRNAs [23].

The requisite dose of RNAi inducers to cause mortality or phenotypic alterations in insects varies with insect species, life stage, the abundance of the target gene transcript and its spatial and temporal expression profiles and according to the delivery method of choice [45]. For example, micro-application of *abnormal wing disc* dsRNA to 5^th^ instar *D*. *citri* nymphs caused significant nymphal mortality and adult wing malformation, and these adverse effects and target mRNA knockdown in ACP were positively correlated with the amounts of dsRNA used [16]. In our study, the efficacy of dsRNA on *D*. *citri* was examined by the addition of different concentrations of *cathepsin D*, *chitin synthase* and *inhibitor of apoptosis* dsRNAs via an artificial diet over time. The results presented here demonstrate clearly the positive correlation between the dsRNA concentrations and mortality for all target genes tested. All the dsRNAs examined here induced higher mortality than the GFP dsRNA controls, suggesting that the respective mRNAs are good RNAi targets for *D*. *citri*. To assess whether RNAi-mediated mortality resulted from a corresponding reduction in *cathepsin D*, *chitin synthase* and *inhibitor of apoptosis* mRNA transcript levels, we examined the expression of these genes using qRT-PCR analysis. The down-regulation of the three target gene transcript levels after 120 h of feeding dsRNAs on artificial diet at 200, 500 and 1000 ng.μL^-1^ was significant compared with GFP dsRNA controls. However, there were no correlations between high dsRNA dosages and reductions in the mRNA expression levels. It is probable that high dsRNAs concentrations are related to the toxic effects of dsRNAs in *D*. *citri*, leading to an increase in psyllid mortality generated from off-targets effects or caused by saturation of the RNAi pathway [42,43]. Similar results were reported for *B*. *tabaci* and *H*. *armigera*, revealing no obvious correlation of increase of dsRNA concentrations with the degree of target mRNA knockdown [25,47].

Using artificial diets is a powerful strategy to screen large numbers of target sequences for RNAi activity, particularly for a small and delicate insects such as adult psyllids, but this method is inefficient for evaluating RNAi effects in nymphs because artificial diets can result in high mortality [52]. To overcome the limitations of artificial diets, a method was modified to target *D*. *citri* mRNAs by dsRNA feeding with *M*. *paniculata* leaflets. This method was used to target three mRNAs by feeding dsRNAs, and we demonstrated that it was efficient because the psyllid mortality in the control treatment (water) was less 30% after 11 days of feeding. Moreover, the dsRNAs of each target and GFP were stable in the dsRNA solution containing the plant leaflet and dsRNAs could be detected in *M*. *paniculata* leaves after 11 days. *D*. *citri* survival rates were reduced in nymphs fed on leaflets exposed to specific dsRNAs compared with the GFP dsRNA control at all times evaluated. However, on the last day of the assay, there was a significant difference between the *chitin synthase* and *inhibitor of apoptosis* dsRNA treatments and GFP dsRNA. To confirm that the psyllid mortality resulted from RNAi effects, the expression levels of each target mRNA were evaluated. *Cathepsin D*, *chitin synthase* and *inhibitor of apoptosis* dsRNAs ingested through *M*. *paniculata* leaves caused significant decreases in corresponding transcript levels in *D*. *citri* compared with the control group. A similar tool was developed to explore the roles of whitefly genes involved in the ecdysone pathway using tomato leaflet-mediated dsRNA feeding methods. The results showed that target mRNA knockdown reduced survival and delayed development of the whitefly during nymphal stages. Moreover, the dsRNAs were stable in the solutions containing the tomato leaflets and in the plant leaves during the five-day experiment [30], as demonstrated in our work, suggesting that this is a useful tool for functional gene discovery studies of sap-sucking insects. Studies of dsRNA stability under different environmental conditions are very important, especially for insect control. For example, *N*. *lugens* and *Ostrinia furnacalis* fed rice and maize irrigated with solutions containing dsRNAs exhibited significantly increased mortality rates. Furthermore, dsRNAs were absorbed by crop roots and stable under environmental conditions, indicating that root dsRNA soaking can be used as a bioinsecticide strategy during crop irrigation [53].

Inhibitor of apoptosis proteins are responsible for several cellular processes, including homeostasis, ubiquitination, intracellular signalling, and cellular division [40]. The RNAi-mediated knockdown of *inhibitor of apoptosis* gene expression resulted in an unambiguous lethal phenotype in the *Dengue virus* and *Yellow fever virus* mosquito vector *A*. *aegypti* [41] and reduction of *Lygus lineolaris* nymph and adult lifespans [54]. Insect chitin synthases are the main enzymes for trachea, cuticle and midgut development [38]. Many studies have shown *chitin synthase* mRNA knockdown in insects. For example, *chitin synthase A* dsRNA delivery by injection and artificial diet containing *Escherichia coli* expressing dsRNAs caused disruption of the *S*. *exigua* larval development, resulting in lethality [27,55]. In addition, microinjection of *chitin synthase 2* dsRNA reduced chitin levels in the midguts of *D*. *virgifera virgifera* [56], and feeding of young tobacco leaves expressing *chitin synthase* dsRNA decreased the net weight of larvae, growth and pupation rates of *H*. *armigera* [57]. *Cathepsin D* knockdown in *B*. *mori* by dsRNA injection inhibited programmed cell death of the larval fat body, resulting in the arrest of larval-pupal transformation because cathepsin D is a metamorphosis-specific proteinase involved in metamorphic events [37].

In conclusion, the development of reliable RNAi techniques for *D*. *citri* provides a starting point for assessing gene function and is an effective way to test and validate possible target sites that can be exploited as new control strategies [14–16]. Our results demonstrate that the oral acquisition of dsRNAs via artificial diets and detached plant leaves causes RNAi effects in different development stages of *D*. *citri*. This finding is important because oral delivery of dsRNA to insects is preferred to use such as pest control, although in some insect species the dsRNA is rapidly degraded in the gut lumen by the action of dsRNAses [31]. Thus, the oral acquisition by *D*. *citri* supports the possibility of potentially using RNAi-based strategies for controlling this very important psyllid.

## Supporting information

S1 Fig *Actin* gene expression stability in different experimental conditions calculated by GeNorm. A: Stability of *D*. *citri actin* gene from feeding assays in artificial diet; B: Stability of *D*. *citri actin* gene from feeding assays in leaflet plant.
(TIF)

S2 Fig Stability of target genes and GFP dsRNAs at different concentrations from artificial diet solutions after 5 days of feeding assays. A: *cathepsin D* dsRNA; B: *chitin synthase* dsRNA; C: *inhibitor of apoptosis* dsRNA; D: *GFP* dsRNA. M: 100 bp ladder; 1: 200 ng.μL^-1^; 2: 500 ng.μL^-1^; 3: 1000 ng.μL^-1^.
(TIF)
