# Supplementary figures and images for: Oral delivery of double-stranded RNAs induces mortality in nymphs and adults of the Asian citrus psyllid, Diaphorina citri
Source: PLoS One. 2017 Mar 10;12(3):e0171847. doi: 10.1371/journal.pone.0171847 (PMC5345766; doi:10.1371/journal.pone.0171847)

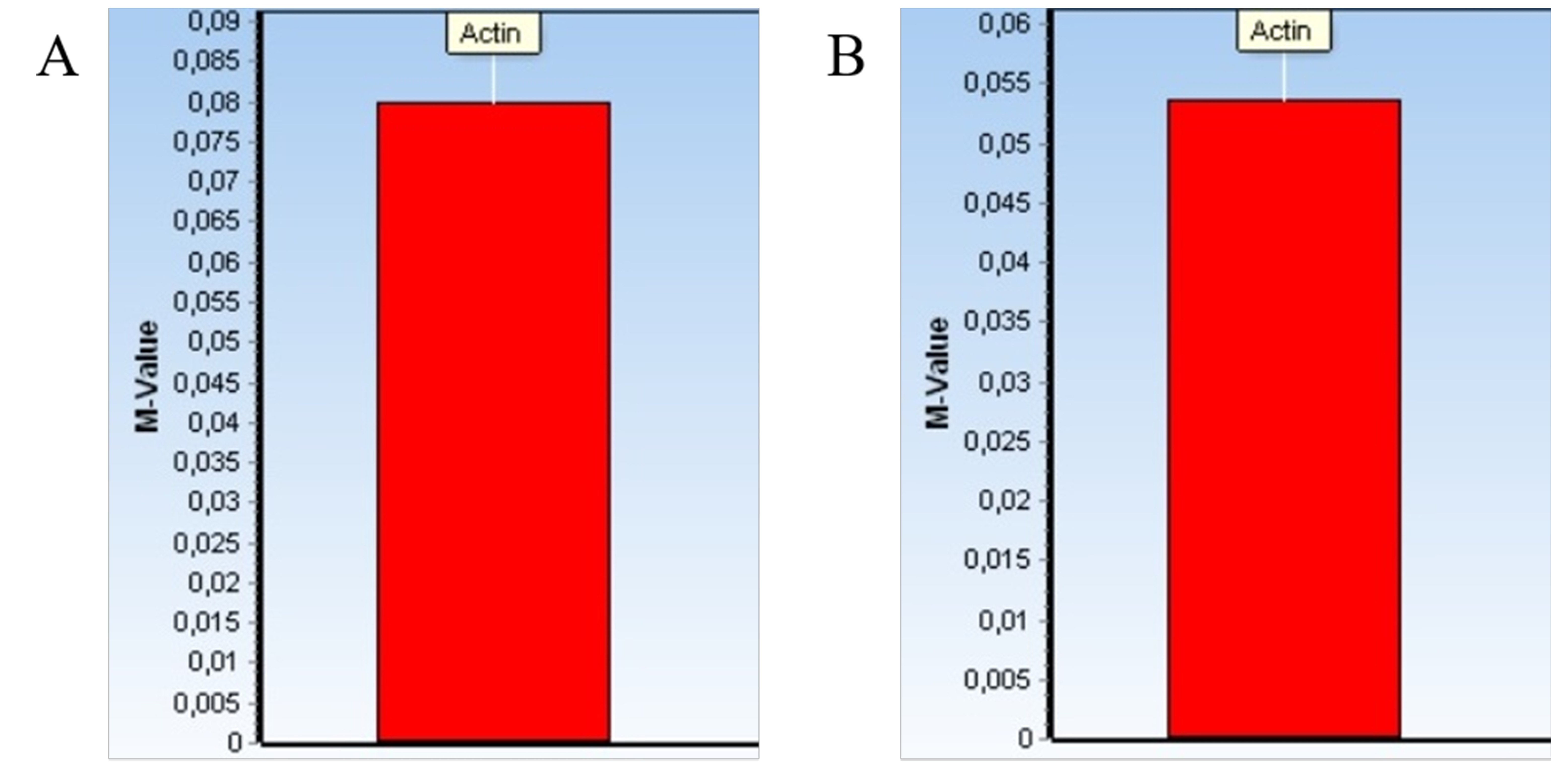

Supplement: S1 Fig — A: Stability of D. citri actin gene from feeding assays in artificial diet; B: Stability of D. citri actin gene from feeding assays in leaflet plant. (TIF) [file pone.0171847.s001.tif]

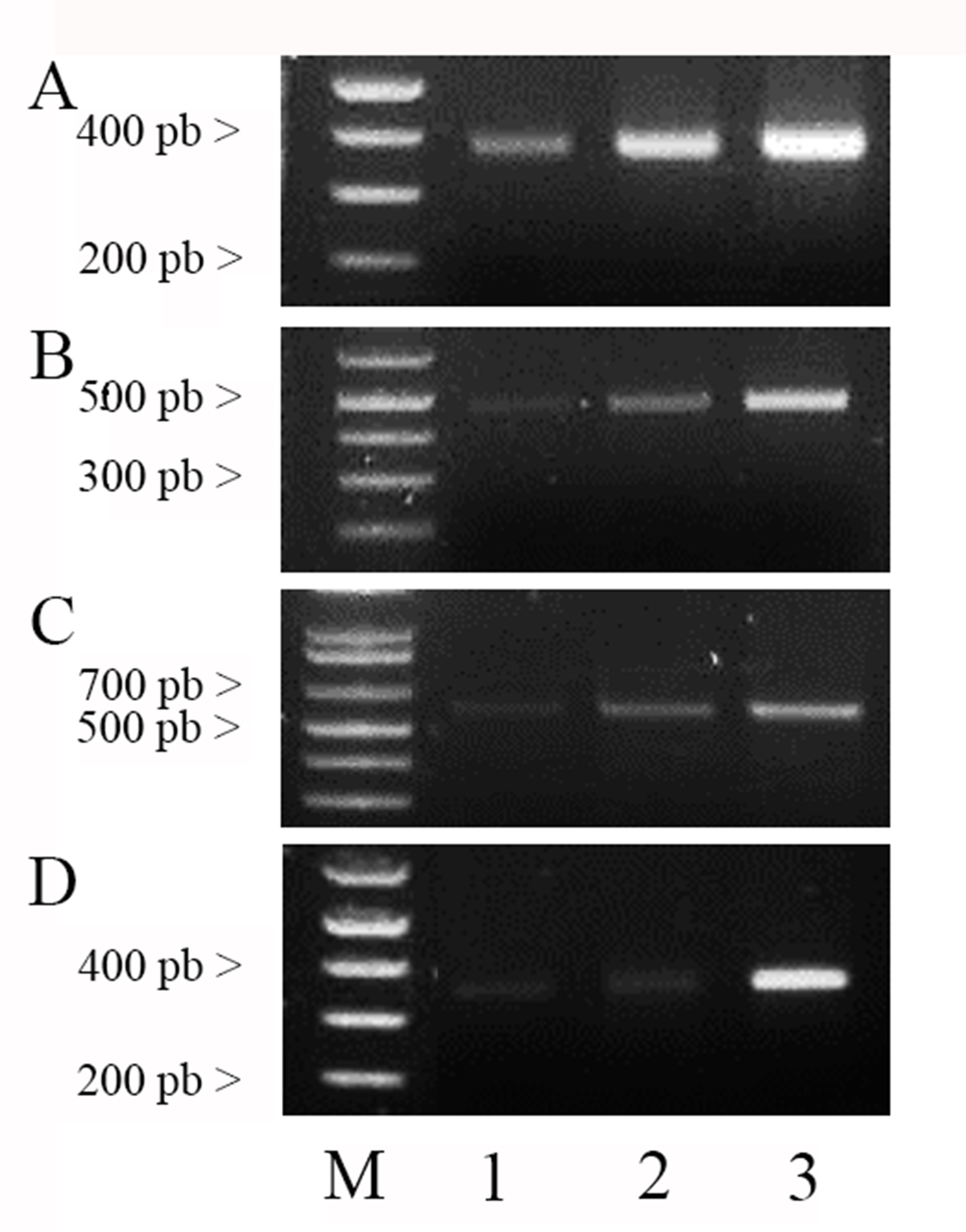

Supplement: S2 Fig — A: cathepsin D dsRNA; B: chitin synthase dsRNA; C: inhibitor of apoptosis dsRNA; D: GFP dsRNA. M: 100 bp ladder; 1: 200 ng.μL-1; 2: 500 ng.μL-1; 3: 1000 ng.μL-1. (TIF) [file pone.0171847.s002.tif]
